# Validation of a measure to assess Post-Traumatic Stress Disorder: a Sinhalese version of Impact of Event Scale

**DOI:** 10.1186/1745-0179-3-4

**Published:** 2007-02-16

**Authors:** Prashantham Baddam John, Paul Swamidhas Sudhakar Russell

**Affiliations:** 1 Christian Counselling Centre, Vellore 632 002, India; 2 Child and Adolescent Psychiatry Unit, Department of Psychiatry, Christian Medical College, Vellore 632 002, India

## Abstract

**Background:**

There is paucity of measures to conduct epidemiological studies related to disasters in Sri Lanka. This study validates a Sinhalese translation of the Impact of Event Scale- 8 items version (IES-8) for use in Sri Lanka.

**Methods:**

This cross-sectional validation study was conducted in the densely populated rural area of Tangalle in the Southern province of Sri Lanka. The English version of the IES-8 after translation procedures in to Sinhalese was administered by trained raters to a community sample of 30 survivors of tsunami aged 13 years and above. Diagnostic accuracy, reproducibility and validity of the translated IES was assessed in terms of sensitivity, specificity, predictive values, likelihood ratios, diagnostic odds ratio, inter-rater reliability, internal consistency, criterion validity and construct validity.

**Results:**

The cut-off score of 15 gave a fair sensitivity (77%) for screening along with other components of diagnostic accuracy. The inter-rater reliability was high (0.89). The internal consistency for the whole scale was high (0.78) with a high face and content validity. The criterion validity was high (0.83) and the construct validity demonstrated the two factor structure documented in the literature.

**Conclusion:**

This study demonstrates that this Sinhalese version of the Impact of Event Scale has sound diagnostic accuracy as well as psychometric properties and makes it an ideal measure for epidemiological studies related to natural and man made disasters in Sri Lanka.

## Introduction

Epidemiological studies exploring psychiatric disorders like Post-Traumatic Stress disorder (PTSD) needs culturally validated measures. Natural and man-made disasters result in stress related disorders among the surviving populations and Sri Lanka in the past had suffered from both kinds of disasters. Post-Traumatic Stress disorder is a well documented outcome of such disasters and although 31,000 people lost their lives in the tsunami that affected Sri Lanka [1] the prevalence of PTSD has not been studied as its aftermath. However, among the survivors of war PTSD had been decribed in 27–72% of the population affected by landmines [2,3] depending on the measure used to define PTSD in these studies. The measures used in these studies have not validated to the Sri Lankan culture and language.

Although there are many measures to study PTSD, the favoured measures to study PTSD in Sri Lanka had been Stress Impact Questionnaire, Harvard Trauma Questionnaire and more recently the Impact of Event Scale [2,4,5]. Among these measures, the Impact of Event Scale (IES) with only 8 items is brief enough to be used in community surveys following major disaster in resource poor countries as well as has excellent, documented psychometric properties [6] and thus makes it the ideal measure for validation to the Sri Lankan culture and language. About 14,8000,000 people in Sri Lanka [7] and another sizable population of 300,000 in Southeast Asia, Middle East, Australia, main land Europe, UK, USA and Canada speak Sinhalese [8]. Many of these countries have endured repeated civil conflicts or terrorism exposing the populations to the stress associated with this violence. Impact of Event Scale has already been validated in Tamil [9] and it only remains to validate IES in Sinhalese. Therefore, the aim of this study is to translate and validate a Sinhalese version of the Impact of Event Scale among those exposed to the tsunami.

## Methods

### Settings and participants

The participants were 30 survivors of the Asian tsunami of 2004 from a densely populated rural area of Tangalle in the Southern province of Sri Lanka. All individuals above 13 years of age and willing to participate in the study were interviewed. They all spoke Sinhalese and majority of them belonged to the fishing community.

### Measures

#### Impact of Event Scale- 8 item version (IES-8)

The 8-item version of Impact of Event Scale has been widely used to study PTSD over the past 20 years and has the intrusive sub-scale as well as avoidance sub-scale with 4 items each. Each item is scored positively, with the levels of endorsement valued at 0, 1, 3, and 5 respectively. A score of 17 or higher was considered a cause for clinical concern [10].

#### DSM-IV-TR clinical interview

The clinical interview was based on the *Diagnostic and Statistical Manual of Mental Disorders, Fourth Edition, Text Revision*. The diagnostic guideline for PTSD (309.81) stipulates that the person should have been exposed to a traumatic event with atleast one symptom from the re-experiencing (intrusion) symptom cluster, three symptoms from avoidance symptom cluster and one symptom from the hyperarousal symptom cluster. These symptoms should have been present for atleast a month resulting in clinically significant distress or impairment in social, occupational, or other important areas of functioning. Those participants who full filled these criteria are diagnosed to have PTSD [11].

### Procedures

The IES in English was translated into Sinhalese and the comparability of content was verified through back-translation procedures by individuals adapt in both languages. The back translated IES with the closest fit to the original tool was selected for validation. The translated version of the IES was administered by the trained raters to a convenient sample of participants in the community exposed to the tsunami to collect the feasibility, reliability and validity data. A qualified psychiatrist with experience in working with people with PTSD interviewed the survivors using DSM-IV-TR guidelines for PTSD as the reference standard. Informed consent was obtained from the participant before the interview or data collection and the research protocol was approved by the Christian Counselling Centre's institutional review board.

### Data analyses

Receiver operating characteristic (ROC) curve was obtained for IES plotting sensitivity against 1 – specificity for every observed cut-off point. We calculated the *predictive values, likelihood ratio *and *diagnostic odds ratio *using the contingency tables for the cut-off point that had the best *sensitivity *and *specificity *for the IES. For *internal consistency*, Cronbach's α coefficient and Spearman's correlation coefficient for the *content validity *were calculated for the two established IES subscales of intrusion and avoidance as well as the total IES score. To determine the *criterion validity *of the IES as a measure of PTSD, the total score and subscale scores of IES was correlated with DSM-IV-TR diagnosis of PTSD. The *Factor structure *of IES was demonstrated by principal components analysis with varimax rotation. Confidence interval was calculated where ever appropriate. Significance was set at P < 0.05, two tailed and data was analysed using SPSS (version 11) software.

## Results

The mean (sd) age of the participants was 37.6 (13.5) years and there both genders were equally distributed in the study sample. The mean (sd) IES score for the sample was 21.6 (7.9).

### Diagnostic accuracy

An IES score of 15 was chosen as the optimal cutoff for screening, as it provided a sensitivity of 77% and specificity of 22% for a DSM-IV-TR diagnosis of PTSD. Higher threshold cutoffs resulted in a loss of sensitivity although there was an appreciable increase in specificity; lower threshold cutoffs resulted in considerable loss of specificity. Considering the need to utilise the IES as a screening tool in the community in post-disaster contexts, the cut-off with better sensitivity was selected. For the cut-off score of 15, the positive predictive value was 0.31 (95%CI = 0.24 to 0.38), negative predictive value was 0.60 (95%CI = 0.25 to 0.87), positive likelihood ratio was 0.93 (95%CI = 0.63 to 1.23) and negative likelihood ratio was 1.3 (95%CI = 0.28 to 5.81).

### Reproducibility

The *inter-rater reliability *calculated by the intra-class coefficient was 0.84 for the whole scale. The inter-rater reliability for the intrusion and avoidance subscales was 0.91 and 0.83 respectively.

### Validity

*Internal Consistency *measured by the Cronbach's α coefficients was high (total score α = 0.78) for the entire IES but was moderate to high for the subscales (intrusion subscale α = 0.43, avoidance subscale α = 0.82) suggesting that the Sinhalese IES has satisfactory internal consistency: The *content validity *calculated with the Spearman's correlations between the subscales (intrusion and avoidance rho = 0.46, P = 0.01). This correlation between intrusion and avoidance subscales suggested that the sub-scales were relatively independent of one another, each of them representing a different type of reaction in the face of stressful events. Also, none of the 8 items was assigned a score of 0 by more than half of the survivors of tsunami in this study reflecting that the content of IES was appropriate for their traumatic experience. The *criterion validity *between the Sinhalese IES and DSM-IV-TR diagnosis of PTSD, calculated with Spearman's correlations were moderate to high at 0.01 level except for the intrusion subscale (intrusion rho = 0.56, P = 0.09; avoidance rho = 0.76, P = 0.009; total rho = 0.83; P = 0.002). The *construct validity *of the Sinhalese IES was determined with factor analysis and we forced the 8 items of the IES into a two-factor structure. Factor loadings 0.50 were considered significant. All these items loaded on to the expected factors distinctively except for item 6 (*Pictures about it popped in to my mind*) which cross-loaded on both the intrusion factor and avoidance factor. Thus item 1, 3, and 7 loaded on factor 1 (intrusion factor), where as item 2, 4 and 8 loaded on factor 2 (avoidance factor). This two-factor structure explained 60.8% of the variance (Table 1). The clinical interview by the psychiatrist using the DSM-IV-TR furnished the comparable construct from a conceptual perspective.

**Table 1:** Factor loadings of the 8-item two-factor structure of a Sinhalese version of IES ^a^

IES items	Factor 1	Factor 2
1. I thought about it when I didn't mean to	0.73^b^	0.08
2. I tried to remove it from my memory	0.07	0.74^b^
3. I had waves of strong feelings about it	0.87^b^	0.13
4. I stayed away from reminders of it	0.37	0.74^b^
5. I tried not to talk about it	0.28^b^	0.62
6. Pictures about it popped in to my mind	0.64^b^	0.53
7. Other things kept making me think about it	0.82^b^	0.12
8. I tried not to think about it	0.35	0.52^b^

Total variance explained (%)	43.2	17.6

IES = Impact of Event Scale. N = 30 tsunami survivors.

^a ^Principal component analysis. Rotation method: Varimax with Kaiser normalization.

^b ^= Loadings > 0.50.

## Discussion

This study followed the criterion referenced validation methodology and assessed the diagnostic accuracy, reliability and validity of a Sinhalese translation of the IES in a sample of participants exposed to a natural disaster.

The various components of diagnostic accuracy have not been studied in the past except for the sensitivity and specificity. For a threshold score of 15 with a sensitivity of 77% as well as a specificity of 22% is suitable for a screening tool and has not been used before. Thus, as the utility of this measure is as a screening tool in different Sinhalese speaking populations this validation serves the purpose even if it means over-diagnosing PTSD because of it low specificity. As the psychometric maturity takes place over time, other parameters like the diagnostic odds ratio will help reduce this false positivity. The intra-class correlation coefficient values of 0.83 to 0.91 show that IES has a high inter-rater reliability, which has not been studied before.

We also have demonstrated that the Sinhalese translation of IES possesses high internal consistency and the α coefficient of 0.78 is comparable with many other studies. However, the internal consistency for the intrusion subscale of 0.43 is lower than in most of the studies and the avoidance subscale value of 0.82 is comparable with the previous works [10,12,13]. The content validity shown by the Spearman's rho of 0.46 is comparable with three other studies [10,12,14]. The criterion validity ranging from 0.56 to 0.83 was much higher than reported [15]. This could be because of the training the raters had before they collected data in which their theoretical knowledge and inter-rater reliability were focused.

Our results replicated a 2-factor structure and it is consistent with the proposed theoretical structure of the scale originally reported [10]. Previous literature has reported different factor structures varying from a 1-factor structure that explained 49% of the variance [16] to a 4-factor structure [17] that explained 63.8% of the variance. This study thus endorses the 2-factor structure, with a better explanation of variance, which can discriminate between stress reactions at different times after the event [8]. The principal component analysis resulted in 3 intrusion and 4 avoidance symptoms, as item 6 (*Pictures about it popped in to my mind*) cross-loaded on both the intrusion factor and avoidance factor. Therefore, our factor structure differed from those in the theoretical model where 4 intrusion and 4 avoidance items (Table 1). This may be because, as in this study where subjects had to recollect the traumatic event that happened over one and a half year, the distinction between intrusion and avoidance distorts over time resulting in one over-all pattern of stress reactions, containing both intrusive and avoidant symptoms as measured with the IES [18]. Also, it should be mentioned that although the study methodology followed the criterion referenced validation and not the norm referenced validation method requiring a relatively smaller sample size, the overall sample size was small.

In conclusion, this Sinhalese version of IES has proven to be a valid measure for posttraumatic stress symptoms in our sample of participants and enhances the ability to assess this disorder among the Sinhalese speaking population to participate in international studies and clinical care.

## Abbreviations

DSM-IV-TR = Diagnostic and Statistical Manual of Mental Disorders, Fourth Edition, Text Revision.

IES = Impact of Event Scale

PTSD = Post-Traumatic Stress disorder

ROC = Receiver operating characteristic

## Competing interests

The author(s) declare that they have no competing interests.

## Authors' contributions

PBJ was involved in the conception, drafting and revising the final draft. PSSR was involved in conception, designing, data analysis and interpretation, drafting and approving the final version.
